# Does a Muscle Fatigue-Inducing Protocol Alter the Magnitude of Jump Inter-Limb Asymmetry in Healthy Adolescents?

**DOI:** 10.5114/jhk/205705

**Published:** 2025-10-24

**Authors:** Joachim D’Hondt, Mara Vanhoudt, Maluta Dani, Laurent Chapelle, Dirk Aerenhouts

**Affiliations:** 1 Movement and Nutrition for Health and Performance (MOVE) Research Group, Department of Movement and Sport Sciences, Faculty of Physical Education and Physiotherapy, Vrije Universiteit Brussel, Brussels, Belgium.

**Keywords:** muscle damage, DOMS, visual analogue scale, jump tests, functional asymmetry

## Abstract

This study examined changes in jump asymmetry in adolescents over a 72-hour period following a muscle fatigue-inducing protocol. Single-leg 10-s hop jump (SL10J), single-leg countermovement jump (SLCMJ), and single-leg horizontal jump (SLHJ) asymmetries were measured at baseline, immediately post-exercise, and at 24, 48, and 72 h post-exercise in 7 female and 16 male adolescents aged 12–18 years. The bioelectrical impedance analysis derived segmental phase angle and ECW/TBW were used as indicators of muscle damage for each leg, while the Visual Analog Scale (VAS) assessed muscle soreness and the bilateral countermovement jump (BCMJ) and the bilateral horizontal jump (BHJ) indicated changes in absolute jump performance. A significant increase in SL10J asymmetry was observed 48 hours post-exercise (p < 0.001), while no significant changes were detected immediately post-exercise or at 24 and 72 h. No significant changes were found in SLCMJ or SLHJ asymmetry at any time point. BIA did not indicate significant muscle damage, though a significant increase in muscle soreness (VAS), peaking at 48 h post-exercise, was recorded. The kappa values indicated slight to moderate agreement for task specificity and slight to substantial agreement for time specificity, with the highest consistency between 24 and 48 h post-exercise for both the SLCMJ and the SL10J. To conclude, asymmetry significantly increased only for the SL10J at 48 h post-exercise, suggesting that repeated jump tests may be more sensitive to fatigue-induced asymmetry than single-jump tests. The substantial kappa values at 24 and 48 h highlight the potential to offer a more robust assessment of asymmetry when recovering from a fatigue-inducing event.

## Introduction

Asymmetry is often defined as the lack of symmetry or equality between halves or sides of the human body and can manifest itself across different dimensions (Bishop et al., 2018). As such, functional asymmetry relates to differences between body sides in performance or function (e.g., side-to-side differences in jump performance), whereas morphological asymmetry expresses variations in size or proportion between limbs (e.g., side-to-side differences in muscle mass) (D'Hondt et al., 2022). Inter-limb asymmetry is presumed to result from the process of lateralisation, in which the dominance of one of the cerebral hemispheres leads to a dominant upper or lower limb (Iskra et al., 2019). Additionally, sport participation has been argued to influence the magnitude of inter-limb asymmetry (Chapelle et al., 2022).

In recent years, inter-limb asymmetry has received significant attention in the context of sport performance and injury risk (Arboix-Alió et al., 2025; D'Hondt et al., 2024; Guan et al., 2022; Lyu et al., 2025). Several studies have demonstrated negative associations between inter-limb jump asymmetry and sport performance (Bishop et al., 2022; Maloney et al., 2017). For example, unilateral drop jump height asymmetries were negatively associated with both change of direction (r = 0.52–0.66, *p* < 0.05) and 10-m (r = 0.52, *p* < 0.05) and 30-m (r = 0.58, *p* < 0.05) sprint performances in male adults and female soccer players, respectively (Bishop et al., 2022; Maloney et al., 2017). In contrast, in another study, vertical jump asymmetries (i.e., single-leg countermovement jump (SLCMJ)) were not significantly (r = −0.091–0.093, *p* = 0.237–0.902) associated with performance related variables (bilateral CMJ (BCMJ), 14- and 18-m sprint, and agility using a cone drill test) in youth basketball players (Dominguez-Navarro et al., 2024). Interestingly, this latter study aimed to examine performance differences across three groups categorized by their magnitude of asymmetry in the unilateral SLCMJ (i.e., 0–9.9%, 10–14.9%, and > 15%) and reported no significant differences in sprint, agility, and bilateral BCMJ performances between groups in both sexes (*p* = 0.101–0.871) (Dominguez-Navarro et al., 2024).

Although there exists some debate on the relationship among inter-limb asymmetry, sport performance and injury risk, it is clear that inter-limb asymmetry is a complex characteristic influenced by various factors (D'Hondt et al., 2024). For instance, previous research reported inter-limb asymmetry to be task-, metric-, direction-, time-, and individual-specific (Chapelle et al., 2021, 2022, 2023; D'Hondt and Chapelle, 2024). Among others, fatigue has been demonstrated to alter the magnitude of inter-limb asymmetry (Heil et al., 2020). More specifically, limbs may respond differently to strenuous exercise with regard to peripheral fatigue, muscle damage and soreness, leading to increased magnitude of inter-limb asymmetry in functional performance measures.

Previous research demonstrated a two-fold increase in the magnitude in SLCMJ peak force asymmetry within 60 min after a game in elite adolescent male soccer players, remaining elevated 48 h post-match (Bromley et al., 2021). In contrast, no significant difference was observed in SLCMJ jump height between pre- and immediately post-match (Bromley et al., 2021). In recreationally active males, Bishop et al. (2021) demonstrated significant increases (*p* < 0.05) in SLCMJ height asymmetry during a repeated sprint protocol (i.e., 6 x 40-m sprint with 20 s of recovery). Meanwhile, a fatiguing protocol targeting the stretch-shortening cycle (14 sets of 10 continuous vertical jumps) in active males did not appear to alter inter-limb asymmetry in ground impact, stiffness or lower-limb coordination during a bilateral step-off landing task (Knihs et al., 2021). However, moderate effect sizes were observed for peak ground reaction force (0.61) and leg stiffness (0.61), suggesting that these latter measures may be influenced by fatigue.

Despite these advancements in knowledge, research on the effects of acute fatigue on inter-limb functional asymmetry remains scarce. Given that peripheral fatigue following stretch-shortening cycle exercise has been reported to be detrimental for sport performance and increase injury risk (Silva-Cavalcante et al., 2019; Verschueren et al., 2020), it is important to examine the possible impact on inter-limb asymmetries under a fatigued condition. Understanding how fatigue interacts with inter-limb asymmetry can help tailor more effective training and rehabilitation protocols aimed at addressing these asymmetries. To achieve this, it is essential to target the same muscle groups involved in the assessment of inter-limb asymmetry. Therefore, the primary aim of this study was to track changes in SLCMJ, single-leg horizontal jump (SLHJ), and single-legged 10-s jump (SL10J) inter-limb asymmetry magnitude from baseline to 72 h following a muscle-fatiguing protocol (i.e., immediately after, and at 24, 48, and 72 h post-exercise) in an adolescent population.

## Methods

### Participants

A total of 23 participants, including 16 males (age = 14.1 ± 1.9 years, body height = 167.3 ± 10.5 cm, body mass = 54.0 ± 9.1 kg, maturity offset = 0.070 ± 1.87 years) and 7 females (age = 15.6 ± 1.3 years, body height = 167.5 ± 7.7 cm, body mass = 61.3 ± 12.8 kg, maturity offset = 0.56 ± 0.96) were recruited through convenience sampling for this observational study. A priori power analysis using G*power 3.1.9.7 (University of Dusseldorf, Germany) demonstrated that, with a medium effect size of 0.25, a power of 0.8 and alpha of 0.05, a sample of 21 participants was required. Inclusion criteria were: (1) adolescents aged between 12 and 18 years, and (2) practicing no more than 3 h per week of any particular sport, excluding elite-level athletes. Participants sustaining an injury within the past 12 months or experiencing pain at the time of testing, were excluded from the study. Participants were instructed to refrain from taking part in other studies involving experimental exercise programs and from engaging in intense physical activities during the follow-up period.

All participants as well as their parent(s) or legal guardian(s) were fully informed about the study procedures and signed written informed consent. The study adhered to the recognized ethical standards and received approval from the Institutional Review Board of the Vrije Universiteit Brussel institution, Brussels, Belgium (approval code: B.U.N. 1432022000252; approval date: 12 April 2023).

### Measures

#### Anthropometrics

Participants' body height (to the nearest 0.1 cm), sitting height (to the nearest 0.1 cm) and body mass (to the nearest 0.1 kg) were measured only at baseline using a stadiometer (SECA 217, Hamburg, Germany) and a digital scale (Omron HN288, Healthcare BV, Hoofddorp, Netherlands), respectively. Leg length was calculated by subtracting sitting height from body height. Based on these assessments, the maturity offset (i.e., years from peak height velocity) was determined using the sex-specific prediction of Mirwald et al. (2002).

#### Muscle Fatigue-Inducing Protocol

The muscle fatigue-inducing protocol involved 10 sets of 10 maximal-effort CMJs, with participants instructed to jump as high as possible at each attempt (Twist and Eston, 2005). A one-minute rest interval between sets was respected. Prior to commencing the protocol, participants performed one practice jump. During each landing, participants were required to adopt a knee joint angle of approximately 90° to absorb the impact. Participants were instructed to keep their hands on their hips throughout each set of 10 jumps.

#### Bioelectrical Impedance Analysis

BIA (Inbody S10, InBody CO., Seoul, Korea) was used at baseline and at 24, 48 and 72 h post-exercise to determine the segmental muscle damage following the muscle fatigue-inducing protocol. More specifically, the ratio of extracellular water / total body water (ECW/TBW) and the phase angle were analysed to estimate the level of segmental muscle damage (da Silva et al., 2023). Before testing, participants were instructed to empty their bladder and to remove all metal-containing objects. The measurements were carried out motionless in a supine position on a non-conductive surface with their arms and legs spread according to the Inbody S10 manufacturer’s guidelines (InBody S10 User’s Manual). Participants had to hold this position for at least five minutes before the start of the measurement. After cleaning the skin with an InBody tissue, 8-point touch type electrodes were attached on the pollex and the digitus medius of the hands and on both sides of the calcaneus bone of the feet (D'Hondt et al., 2022).

#### Muscle Soreness

The Visual Analogue Scale (VAS) was used as a subjective measure of muscle soreness prior to each test session, following the performance of a squat to approximately 90° of knee flexion with hands placed on the hips. Participants had to indicate their muscle soreness on a 100 mm horizontal line with “no muscle soreness/pain” and “too sore to move/unbearable pain” displayed on the left and right extremity of the scale, respectively. A pain-rating index was calculated from each VAS-score, with the intensity of pain and muscle soreness quantified by the distance (in mm) from the left-hand side of the scale.

#### Jumping Tests

All jumping tests were performed during each testing session. Before the start of each jumping assessment, participants completed a standardized warm-up, consisting of two sets of 10 squats, two sets of 10 toe raises, and three sets of each test, with a 20-s rest interval between sets. A minimum of 3 min of recovery was provided between the warm-up and the test protocol.

Each jump test was conducted three times, with 30 s of recovery between subsequent attempts. When a jump did not meet the specified criteria, the participant repeated the trial after a 30-s rest interval. All tests were performed barefoot. Verbal encouragement by the same researchers was provided to ensure maximum effort. All unilateral tests were conducted on both legs. For the SLCMJ, the BCMJ, the SLHJ and the bilateral horizontal jump (BHJ), the highest or the furthest attempt was used for analysis, whereas for the SL10J, the average jump height was calculated and used for analysis. Jump height during the vertical jump tests was measured to the nearest 0.1 cm using an Optojump Next system (Micro-grate, Bolzano, Italy), whereas all horizontal jump distances (i.e., distance covered from a marked starting line to the heel of the participant’s landing foot) were measured to the nearest 0.1 cm using a 3-m-long tape that was placed firmly on the ground surface.

#### Single-leg Countermovement Jump

To isolate the contribution of the lower limbs, participants were instructed to perform the jumps without an arm swing, keeping their hands on the hips throughout the movement. The test leg was required to be fully extended at the start of the test, while the non-testing leg was slightly bent with the foot hovering at the mid-shin level. Additional swinging of the non-testing leg was prohibited during the test trials. From this position, participants executed a maximal vertical jump, initiated by a self-selected countermovement depth followed by a rapid vertical acceleration. The tested leg remained fully extended during the flight phase of the jump before landing (Chapelle et al., 2023).

#### Bilateral Countermovement Jump

This test followed similar instructions as the SLCMJ (Chapelle et al., 2023). However, for the BCMJ, participants were required to push off and land on both legs simultaneously.

#### Single-Leg Horizontal Jump

Participants had to position behind a pre-marked starting line with the hallux. The starting position of the body was identical to the SLCMJ. Subsequently, participants were instructed to jump as far as possible in the forward direction. Successful test execution was confirmed if the participant kept their hands on the hips and maintained balance without altering the dominant foot position for at least 2 s after landing.

#### Bilateral Horizontal Jump

The BHJ was performed using the same setting, technique and instructions as for the unilateral horizontal jumps. Instead of jumping on one leg, participants had to push off and land on both legs simultaneously.

#### Single-Legged 10-s Jump

The starting position and instructions for this test were identical to those for the SLCMJ. Participants executed a rapid unilateral countermovement into a self-selected depth, followed by a maximal vertical jump and continued jumping for duration of 10 s. Participants were instructed to jump as high as possible while minimizing ground contact time and maintaining control upon landing. Each leg was tested in a single trial.

#### Asymmetry Index

Based on the results from the jump tests, scores for inter-limb asymmetry were computed. For this, the percentage difference method was used: % asymmetry = ([dominant performance value – non-dominant performance value] / dominant performance value)*100 (Bishop et al., 2018).

### Design and Procedures

#### Timeline

A total of five testing sessions were conducted over four days. Participants were assessed at baseline, immediately after the fatiguing protocol, and at 24 h, 48 h, and 72 h after the fatiguing protocol. A standardised measurement procedure was employed throughout all test occasions.

### Statistical Analysis

Statistical analyses were performed using SPSS software version 29, with the significance level alfa set at 0.05. Normality of the data was assessed using the Shapiro-Wilk test, while sphericity was evaluated with the Mauchly's test. A one-way repeated measures mixed design ANOVA was conducted for SL10J asymmetry, with measurements taken at five time points: before the muscle fatigue-inducing protocol (T0), immediately after (T1), 24 h after (T2), 48 h after (T3), and 72 h after (T4) the muscle fatigue-inducing protocol. Since Pearson correlation indicated that SL10J inter-limb asymmetry was not significantly associated with maturity offset across all test occasions (*p* = 0.028–0.783), maturity offset was not used as a covariate in the analysis. Since the box’s Test of Equality of Covariance Matrices was significant (*p* = 0.013, F = 2.019), Pillai’s Trace was used for the multivariate tests, and Greenhouse-Geisser corrections were applied to the degrees of freedom for the within-subjects effects. Effect sizes were measured using partial eta squared (ηp^2^) and interpreted as small (ηp^2^ = 0.01– 0.06), medium (ηp^2^ = 0.06–0.14), and large (ηp^2^ ≥ 0.14) (Cohen, 1988). Since SLCMJ asymmetry and SLHJ asymmetry were not normally distributed, a Friedman test was used for these specific tests. Kendall's W was calculated as the effect size for the Friedman tests (small = 0.1–0.3, medium = 0.3–0.5, large > 0.5) (Cohen, 1988). Friedman and Wilcoxon tests were applied to measure the changes in muscle soreness (i.e., VAS-score), muscle damage (i.e., ECW/TBW and phase angle) and jump performances (i.e., SLCMJ, BCMJ, SLHJ, BHJ and SL10J) over time. Kappa coefficients were calculated to evaluate the consistency in which limb performed better across tests (i.e., task specificity) and to assess the consistency of which limb displayed the highest jump performance over time (i.e., time specificity). Kappa coefficients were classified as poor (≤0), slight (0.01–0.20), fair (0.21–0.40), moderate (0.41–0.60), substantial (0.61–0.80), almost perfect (0.81–0.99), or perfect (1.00) (Viera and Garrett, 2005).

## Results

Measures for muscle soreness, muscle damage and jump performance at the different time points are presented in Table 1.

**Table 1:** Mean muscle soreness, muscle damage, jump height and jump distance; results presented as means ± standard deviation (SD).

	PRE	POST	24h POST	48h POST	72h POST
Muscle soreness					
VAS-score (mm)^#^	0.4 ± 0.7	2.6 ± 1.5^a^	3.6 ± 2.1^a, b^	14.5 ± 2.0^a, b^	2.6 ± 1.4^a^
Muscle damage (Bioelectrical impedance analysis)					
Phase angle, right (°)	6.7 ± 0.8		6.8 ± 0.9	6.6 ± 0.7	6.5 ± 0.8^a^
Phase angle, left (°)^#^	6.7 ± 0.9		6.8 ± 0.9	6.6 ± 0.8	6.5 ± 0.8^a^
ECW/TBW, right	0.4 ± 0.0		0.4 ± 0.0	0.4 ± 0.0	0.4 ± 0.0
ECW/TBW, left	0.4 ± 0.0		0.4 ± 0.0	0.4 ± 0.0	0.4 ± 0.0
Jump performance					
SLCMJ, right (cm)^#^	14.4 ± 3.9	14.2 ± 3.6	14.5 ± 3.5	14.7 ± 3.3	15.2 ± 3.2^a, b^
SLCMJ, left (cm)	15.1 ± 3.4	14.6 ± 3.2	14.6 ± 3.3	15.7 ± 3.4	15.4 ± 3.6
BCMJ (cm)	30.3 ± 5.6	30.2 ± 5.5	29.9 ± 5.9	29.0 ± 5.5^a^	30.0 ± 5.6
SLHJ, right (cm)^#^	155.3 ± 24.1	153.0 ± 24.8	157.5 ± 25.5	156.3 ± 24.6	157.0 ± 27.0
SLHJ, left (cm)	156.4 ± 25.7	154.6 ± 25.8	158.1 ± 27.3	159.1 ± 29.0	158.6 ± 25.7
BHJ (cm)	182.9 ± 26.6	179.4 ± 27.2	186.1 ± 25.2^b^	181.4 ± 27.2	187.2 ± 26.3
SL10J, right	12.0 ± 2.9	10.3 ± 2.5^a^	10.4 ± 2.6	9.8 ± 2.2^a^	9.3 ± 2.3^a^
SL10J, left	11.2 ± 2.9	9.9 ± 2.3^a^	10.5 ± 2.4^b^	14.8 ± 3.3^a, b^	9.5 ± 2.3^a^
Asymmetry scores					
SLCMJ %^#^	9.8 ± 9.7	7.3 ± 6.6	10.6 ± 7.0	10.2 ± 7.6	9.9 ± 6.8
SLHJ %^#^	4.4 ± 4.3	5.6 ± 2.8	4.3 ± 4.1	5.2 ± 4.2	4.2 ± 4.87
SL10J %	8.4 ± 7.1	11.1 ± 6.9	10.5 ± 7.5	32.1 ± 11.5 ^a, b^	11.6 ± 8.1

VAS: visual analogue scale, ECW/TBW = extracellular water / total body water ration, SLCMJ = single-leg countermovement jump, BCMJ = bilateral countermovement jump, SLHJ = single-leg horizontal jump, BHJ = bilateral horizontal jump, SL10J = single-legged 10-s jump, ^a^ significantly different from T0 (p < 0.05), ^b^ significantly different from T1 (p < 0.05), note: normally distributed data were analysed using one-way repeated measures ANOVA, whereas non-normally distributed data marked with a ^#^ were analysed using Friedman and Wilcoxon tests

**Figure 1:**
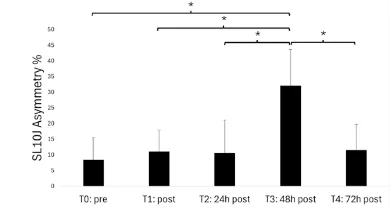
Inter-limb asymmetry values from the single-leg 10-s countermovement jump with * indicating a *p* value < 0.001.

One-way repeated measures ANOVA revealed a significant main effect of the within-subjects factor indicating a significant difference in the magnitude of inter-limb SL10J asymmetry over time (F = 21.581, *p* < 0.001, ηp^2^ = 0.820). Pairwise comparisons demonstrated that the magnitude of inter-limb asymmetry at T3 was significantly higher (*p* < 0.001) than the magnitude of asymmetry measured at any other time points (Figure 1). In contrast, no significant differences in the magnitude of asymmetry were observed between the remaining time points (*p* = 0.124–761).

Friedman test results revealed no significant differences in the asymmetry magnitude of the SLCMJ (*p* = 0.714, W = 0.023) and the SLHJ (*p* = 0.468, W = 0.039) between time points.

**Figure 2:**
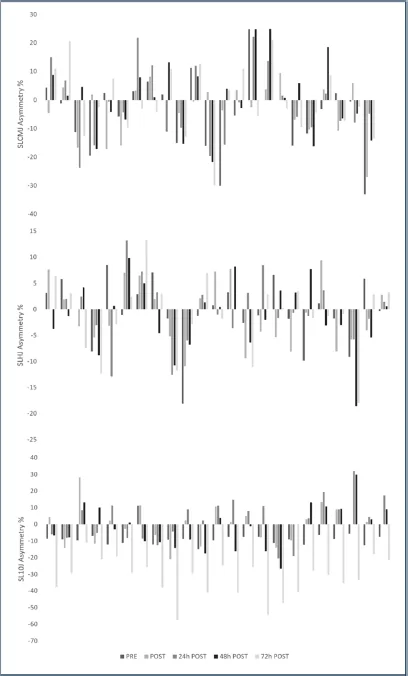
Individual (N = 23) inter-limb asymmetry scores during the 5 test occasions for single-leg countermovement jump (SLCMJ), single-leg horizontal jump (SLHJ) and single-legged 10-s jump (SL10J) tests with positive values indicating greater right leg jump height and negative values indicating greater left leg jump height.

Kappa values were poor to substantial (κ = −0.137–0.732) regarding time-specificity (Table 2) and poor to moderate for task-specificity (κ = −0.123–0.493) (Table 3). All individual inter-limb asymmetry data for the SLCMJ, the SLJH and the SL10J during the five specific test occasions are displayed in Figure 2.

**Table 2:** Time-specificity of functional asymmetry: Kappa coefficients (κ) indicating the agreement in directionality on which limb the highest jump was performed across test moments.

	T0–T1	T0–T2	T0–T3	T0–T4	T1–T2	T1–T3	T1–T4	T2–T3	T2–T4	T3–T4
SLCMJ										
Kappa value	0.045	0.649	0.481	0.298	−0.120	−0.137	−0.120	0.662	0.270	0.324
Level of agreement	Slight	Subst.	Mod.	Fair	Poor	Poor	Poor	Subst.	Fair	Fair
SLHJ										
Kappa value	0.388	0.045	0.045	0.219	0.481	0.135	0.481	−0.045	0.303	0.129
Level of agreement	Fair	Slight	Slight	Fair	Mod.	Slight	Mod.	Poor	Fair	Slight
SL10J										
Kappa value	0.473	0.135	0.052	0.219	0.324	0.253	0.225	0.732	0.213	0.298
Level of agreement	Mod.	Slight	Slight	Fair	Fair	Fair	Fair	Subst.	Fair	Fair

SLCMJ = single-leg countermovement jump, SLHJ = single-leg horizontal jump, SL10J = single-legged 10-s hop jump, Subst. = Substantial, Mod. = Moderate, note: the level of agreements classified according to the Viera and Garret (2005): poor (< 0.00), slight (0.00–0.20), fair (0.21–0.40), moderate (0.41–0.60), substantial (0.61–0.80), almost perfect (> 0.80)

**Table 3:** Task-specificity of functional asymmetry: Kappa coefficients (κ) indicating the agreement in directionality on which limb the highest jump was performed across tasks per test moment.

	T0	T1	T2	T3	T4
SLCMJ x SLHJ					
Kappa value	0.129	−0.038	0.397	0.213	0.052
Level of agreement	Slight	Poor	Fair	Fair	Slight
SLCMJ x SL10J					
Kappa value	0.477	−0.120	0.493	0.195	0.052
Level of agreement	Moderate	Poor	Moderate	Slight	Slight
SL10J x SLHJ					
Kappa value	−0.394	0.195	0.038	−0.053	0.477
Level of agreement	Faire	Slight	Slight	Poor	Moderate

SLCMJ = single-leg countermovement jump, SLHJ = single-leg horizontal jump, SL10J = single-legged 10-second hop jump, note: the level of agreements classified according to the Viera and Garret (2005): poor (< 0.00), slight (0.00–0.20), fair (0.21–0.40), moderate (0.41–0.60), substantial (0.61–0.80), almost perfect (> 0.80)

## Discussion

The aim of this study was to assess changes in functional inter-limb asymmetry magnitude in adolescents over a 72-h period following a muscle fatigue-inducing protocol. Results showed a significant increase in SL10J inter-limb asymmetry at 48 h post-exercise, with no notable changes detected immediately after the protocol or at 24 and 72 h post-exercise. Moreover, no significant variations in SLCMJ or SLHJ inter-limb asymmetry were observed across the different testing sessions. While muscle fatigue may increase the magnitude of SL10J inter-limb asymmetry, BIA findings suggest that the muscle-fatiguing protocol involving 10 sets of 10 maximal vertical jumps may not have induced any considerable muscle damage to evoke detectable changes in SLCMJ and SLHJ inter-limb asymmetry.

Mean inter-limb asymmetry scores ranged from 9.8% to 10.6% for the SLCMJ, 4.2% to 5.6% for the SLHJ, and 8.4% to 32.1% for the SL10J. Intuitively, it seems reasonable to assume that the magnitude of jump asymmetry would increase following a muscle fatiguing protocol, as fatigue and recovery influence each limb differently. This assumption is supported by previous research from Fort-Vanmeerhaeghe et al. (2023) who showed significant increases in SLCMJ asymmetry (*p* = 0.033) after a 30–15 intermittent fitness test in youth female team sport athletes. Similarly, Kons et al. (2022) reported significantly higher asymmetry scores for peak force, peak power and mean power (*p* < 0.05) during a SLCMJ following a stretch-shortening cycle fatiguing-protocol in judo athletes. However, some studies have found no significant pre-post differences or even reduced inter-limb asymmetry magnitude in a fatigued state (Girard et al., 2017; Vial et al., 2023). For example, Vial et al. (2023) observed greater kinematic inter-limb asymmetry during non-fatigued sprinting than during fatigued sprinting in soccer players, with the dominant leg producing greater propulsive impulse and the non-dominant leg exhibiting higher vertical impulse, contributing to higher inter-limb asymmetry.

In our study, only a significant increase in asymmetry magnitude was observed for the SL10J test at 48 h post-protocol. Previous research indicated that DOMS tends to peak 24 to 72 h post-exercise before gradually diminishing (Lewis et al., 2012). Given that exercise-induced fatigue can reduce the level of voluntary muscle activation and influence muscle activation patterns (Barber-Westin and Noyes, 2017), it is possible that the weaker limb is more affected in a fatigued state compared to the stronger limb, thereby increasing the magnitude of inter-limb asymmetry. In contrast to the significant increase in SL10J asymmetry 48 h post-protocol, our results showed no significant difference in SLCMJ and SLHJ asymmetry magnitude following a muscle fatiguing protocol. This is also in contrast to previous findings where significant increases in SLCMJ asymmetry magnitude were observed after a repeated sprint protocol (6 x 40-m sprints with 20 s of recovery) in recreationally active males and a soccer match in professional male players (Bishop et al., 2021; Bromley et al., 2021). A key factor in this discrepancy may be the differences in fatiguing protocols. Both Bishop et al. (2021) and Bromley et al. (2021) induced fatigue through repeated sprints, either directly or indirectly via a soccer match, likely imposing a greater load on the body than the moderate-fatigue repeated jump protocol used in our study. This is further supported by the higher asymmetry magnitude, reaching up to 14.67%, observed in a comparable group of participants (i.e., recreationally physically active males) (Bishop, et al., 2021). Using a similar fatiguing protocol, (i.e., 14 sets of 10 continuous jumps), Knihs et al. (2021) reported no significant changes in ground impact, stiffness and lower-limb coordination asymmetries during a landing task. However, medium effect sizes were observed for increased ground reaction force and leg stiffness asymmetries, highlighting the metric-specific nature of inter-limb asymmetries. These results suggest that assessing only jump height asymmetry may overlook other jump performance-related variables such as ground reaction force and leg stiffness that could be altered by fatigue.

Another potential reason for this discrepancy could be attributed to the intensity of our muscle-fatiguing protocol. While previous research has shown this protocol to effectively induce muscle damage (Twist and Eston, 2005), our study did not observe clear muscle damage through BIA, except for a small decrease in the phase angle 72 h post-protocol. Moreover, no clear differences in absolute single jump performances were apparent post-protocol compared to baseline, indicating that the muscle fatigue-inducing protocol was not sufficiently effective in inducing the necessary fatigue for these specific jump tests. This may be attributed to the relatively low average jump heights of 30 cm as observed in the present study. Given that our study population consisted of non-athletes, lower jump heights were expected, which likely reduced the eccentric load to absorb landing impact and, consequently, muscle damage. However, in contrast to these objective measures, the VAS-scale in the present study showed significant increases in muscle soreness from 24 to 72 h post-exercise. Based on our findings, BIA and bilateral jumps may not be sensitive enough to detect muscle damage induced by the fatigue protocol when compared to subjective measures such as the VAS. Therefore, we recommend that future research on muscle fatigue and inter-limb asymmetry incorporates the combination of objective measures (e.g., BIA, biomarkers or jump performance data) and subjective assessments (e.g., VAS) to more accurately evaluate the effectiveness of fatigue- inducing protocols, rather than relying on a single type of assessment.

Although inter-limb asymmetry did not significantly change across test occasions for the single jump tests, it is important to note that similar results were observed in a recent study by Guan et al. (2021). In their study, a significant increase in inter-limb asymmetry was observed in repeated jump tests (i.e., triple jump) in a fatigued state, but no significant increase was seen in single jump tests (i.e., SLCMJ and SLHJ). These findings suggest that the repeated jump tests may be more sensitive to fatigue compared to single jumps. This may be due to longer duration stressing other metabolic systems (i.e., for ATP restoration, buffering capacity, etc.), and greater complexity of the neuromotricity including the repeated need for deceleration, stabilisation and subsequent concentric power generation (Cuthbert et al., 2021). Given that repeated jumps appear to be more sensitive to fatigue and display higher ecological validity in (team) sports due to their specific movement requirements (Bishop et al., 2021), and based on our and previous study (Guan et al., 2021) results, we recommend practitioners to use repeated jump tests when measuring inter-limb asymmetry in a fatigued state.

According to previous research, inter-limb asymmetry, particularly at the lower limb level, is both task and time specific (Chapelle et al., 2023; D'Hondt and Chapelle, 2024). Our study showed consistent results with slight to moderate kappa values for task specificity, indicating a low agreement in the direction of asymmetry across jump tests. For time specificity, the kappa values ranged from slight to substantial. Notably, substantial kappa values were observed between 24 and 48 h post-protocol for the SLCMJ and the SL10J. Although future research is needed to confirm this hypothesis, our results suggest that true limb dominance could potentially be more easily detected when the limbs are in a fatigued state resulting in more robust asymmetry scores.

## Limitations

As a first limitation, the fatiguing-protocol used—10 sets of 10 maximum vertical jumps—did not appear to be sufficiently strenuous to induce significant muscle damage or fatigue, as indicated by the BIA measures, and absolute bilateral jump performance. Moreover, while all participants underwent the same muscle fatigue-inducing protocol, it is possible that this protocol did not elicit a uniform level of fatigue across different individuals. Factors such as the fitness level, psychosocial well-being, and the recovery state can significantly influence how individuals perceive and respond to physical stress, possibly contributing to variability in fatigue and inter-limb asymmetry (Smeets et al., 2019). This high inter-individual variability suggests that some participants may exhibit higher changes in inter-limb asymmetry magnitude than others under fatigued conditions. Therefore, practitioners should assess changes in asymmetry on an individual basis and should consider employing protocols that elicit both acute and prolonged responses to fatigue (Fort-Vanmeerhaeghe et al., 2023; Vermeulen et al., 2024). Second, our assessment of functional asymmetry was based solely on jump distance or jump height during unilateral jump tests. Previous research has shown that relying solely on jump distance can obscure potential differences in jumping technique (Kotsifaki et al., 2020). To address this, future studies should consider employing a force plate to capture a more comprehensive range of biomechanical variables. Finally, our sample consisted of recreationally active individuals rather than trained athletes. This limits the generalizability of the findings to athletic populations. Future research should include homogenous samples of athletes to enhance the relevance and applicability of results to sport-specific contexts.

## Conclusions

In summary, our findings showed that inter-limb asymmetry in the SL10J peaked 48 h after the protocol and returned to baseline levels by 72 h. In contrast, this muscle fatigue-inducing protocol did not significantly alter the magnitude of asymmetry in the SLCMJ or the SLHJ across different testing sessions. Although the muscle fatigue-inducing protocol was effective in increasing muscle soreness but not in inducing substantial muscle damage, these findings suggest that repeated jump tests may be more sensitive to fatigue than single jump tests when assessing inter-limb asymmetry. Additionally, kappa values indicated that while there was slight to moderate agreement regarding task specificity, the direction of asymmetry was notably more consistent over time between 24 and 48 h post-exercise, highlighting the potential for more robust asymmetry detection when recovering from a fatigue-inducing event.
